# Analysis of causes of death using verbal autopsies and vital registration in Hidalgo, Mexico

**DOI:** 10.1371/journal.pone.0218438

**Published:** 2019-07-03

**Authors:** Dolores Ramirez-Villalobos, Andrea Leigh Stewart, Minerva Romero, Sara Gomez, Abraham D. Flaxman, Bernardo Hernandez

**Affiliations:** 1 Instituto Nacional de Salud Pública, Cuernavaca, México; 2 Institute for Health Metrics and Evaluation, University of Washington, Seattle, Washington, United States of America; 3 North Carolina Farmworker Health Program, Office of Rural Health, Raleigh, North Carolina, United States of America; 4 Department of Health Metrics Sciences, University of Washington, Seattle, Washington, United States of America; The George Institute for Global Health, UNSW, AUSTRALIA

## Abstract

**Introduction:**

Verbal autopsy (VA) is a useful tool for evaluating causes of death, especially in places with limited or no vital registration systems. The Population Health Metrics Research Consortium (PHMRC) developed a validated questionnaire and a set of automated methods to determine the cause of death from a VA. However, the application of these methods needs to be tested in a community environment.

**Objective:**

To estimate cause-specific mortality fractions (CSMFs) using VAs and compare them against those obtained in the vital statistics of the state of Hidalgo, Mexico.

**Methods:**

A random sample of deaths occurred in 2009 was selected from vital statistics in the state of Hidalgo. The full PHMRC validated VA instrument was applied to the relatives of the deceased, and the cause of death was determined using Tariff's automated method. The causes of death were grouped into 34 causes for adults, 21 for children and 6 for newborns. Results were compared with cause of death on death certificates for all deaths.

**Results:**

A total of 1,198 VAs were analyzed. The Tariff method was not able to assign a cause of death in only 9% of adults, 2% of children and 7% of neonatal deaths. The CSMFs obtained from the Tariff method were similar in some cases to those of vital statistics (e.g. cirrhosis), but different in others (e.g. sepsis).

**Conclusion:**

The application of VAs in a community sample, analyzed with the Tariff method, allowed assigning a cause of death to most of the cases, with results similar to those of vital statistics for most conditions. This tool can be useful to strengthen the quality of vital statistics.

## Introduction

Vital statistics are essential to guide the actions of health systems. Developing countries face persistent problems with the recording and classification of causes of death, especially in rural communities where most deaths are not certified by physicians or the person certifying the death is not the treating physician. [1–3] Mexico is considered as a country with high quality reporting of vital statistics, [4–5] but there is still room for improvement. [6] The compilation of vital statistics in Mexico relies on information from death certificates, [7] with the participation of the Ministry of Health and the INEGI (National Institute of Statistics and Geography). [8]

Verbal autopsy (VA) is a technique for determining the cause of death based on a questionnaire applied to family members or people who were in contact with the deceased. The VA reconstructs the history of a person from illness to death. This is a very useful epidemiological surveillance tool. The VA allows the identification of causes of death in places with non-existent, incomplete, or unreliable death records. In some regions of the world it is the only method available to calculate the distribution of causes of death. [9–11] The VA assumes the possibility of identifying the cause of death based on information provided by an informant who had contact with the deceased person. It is therefore important to use a validated instrument. The validity of VA depends on the type of illness leading to death, the characteristics of the deceased, and other factors related to the classification of causes of death, as well as the design, questionnaire content, and field procedures. [12–14] The Population Health Metrics Research Consortium (PHMRC) developed a VA instrument along with automated methods for determining cause of death through VA. This instrument and the automated methods have been validated with a sample of hospital deaths comparing its performance at the individual and population level vs. the cause of death stated in medical records. [15–18] Now, it is important to test it with deaths occurring in health units or elsewhere, where certification problems may exist.

The objective of this study is to assess the causes of death by collecting VAs and analyze them using the Tariff method (an automated method to assign cause of death) in a community sample of deaths in the state of Hidalgo, Mexico, and to compare the cause specific mortality fractions (CSMFs) obtained by this method vs. the CSMFs obtained from vital statistics of that state.

## Methodology

### Study design

This study is part of the verbal autopsies study conducted by the PHMRC, [16] which included the validation of a VA questionnaire in Tanzania, India, the Philippines, and Mexico, the development of automated methods for assigning causes of death, and the application of VA in community samples in the Philippines and Mexico.

### Population and sample

The study included all deaths identified in vital statistics from the state of Hidalgo, Mexico, that occurred between January and December of 2009, whose registry specified the state of Hidalgo as the habitual place of residence of the deceased person.

**Fig 1:**
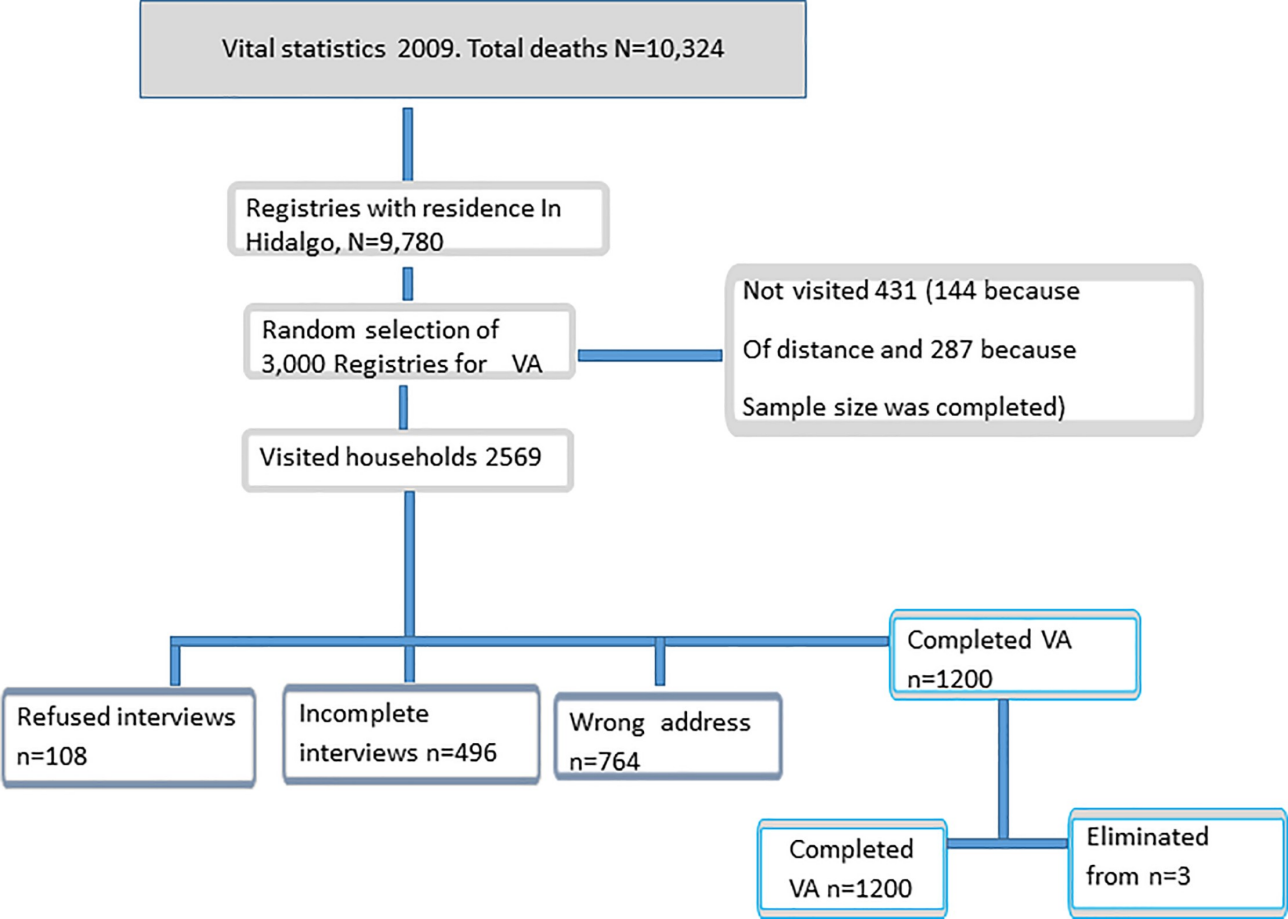
Verbal autopsy sample selection, Mexico, Hidalgo 2009.

The sample integration process is presented in (Fig 1). A sample size of 1200 community VAs was proposed following the design proposed by PHMRC. A total of 10,324 deaths were identified through vital statistics from the state of Hidalgo during 2009. Of these, only those of residents of the state of Hidalgo were eligible for the study (n = 9,780).

Subsequently, assuming a non-response rate of 50%, a total of 3,000 deaths were selected to be included in the study, in sites with larger populations and which register more than 17% of the total of the deaths in the state of Hidalgo. Once the sites were selected, three age groups were randomly sampled: 0–14, 15–59 and 60 and older. The cases were distributed in 330 communities in 13 sites.

### Instruments

We used the full PHMRC VA instrument. The instrument used for the collection of VA information was based on the instrument proposed by WHO, [19] which in turn builds on the work of Chandramohan, et al. for adult deaths and Anker et al. for neonatal and infant mortality. [12] [20] The PHMRC instrument is structured into three separate modules for adults, children and newborns. All interviewees also receive a general information questionnaire on the socio-demographic characteristics of the informant and the event. [16]

### Procedure

Once the sample was selected, all death certificates selected for the study were provided with the address for VA interviews. The VAs were applied to the relative who took care of the patient at home, between May and November of 2011, by interviewers of both sexes who had no knowledge of the cause of death and who were trained in the management of thanatology and emotional support to the informants. All data were collected on paper by an interviewer in a face to face interview.

The interviewers visited the homes of the randomly selected cases from 13 sites, until meeting the target sample of 1200 VAs, requiring a visit to 2,569 homes of the 3,000 initially selected. Incomplete interviews determined when the informant was not found in their home after three visits; informants refusing to participate in the study were determined as denied interviews; wrong address was determined when it was not possible to find the home in the community, neither with the aid of neighbors nor in adjacent sites. Each interview lasted an average of 45 minutes. A simultaneous interpreter was used when necessary, such as when the subject spoke an indigenous language.

The research protocol for this study was approved by the ethics and research committee of the National Institute of Public Health and the health authorities of the state of Hidalgo. All information was obtained with the prior written informed consent of the participants. Given the sensitivity of the interview topic, emotional support was offered to informants.

### Cause of death determined through VAs

Once the VAs were compiled, the information was entered into a database. Causes of death were assigned for each VA using the Tariff method. [21–22] In summary, this method assigns a score (*Tariff score*) [23] to each cause of death according to the responses of the informants for each indicator of the VA questionnaire. This method allows us to determine causes of death in a list of 34 causes for adults, 21 for children and 6 for newborns, excluding stillbirths. The information was analyzed using the Tariff method as available in the SmartVA software, and including the option to use the information from the open narrative questions to assign the cause of death. [24]

### Causes of death in vital statistics

To determine the basic cause of death according to vital statistics, the official coding of the basic cause of death for each of the deaths included in this study was obtained from the mortality database of the INEGI (National Institute of Statistics and Geography) and the Ministry of Health and grouped according to the classification of causes of death used in the PHMRC study. Considering that this database included 19% of cases with poorly defined causes of death according to the classification of the 2010 Global Burden of Disease study, the cause of death was reassigned following the guidelines of that study. [25] We used the redistribution method developed by Naghavi and cols [26]—This method reclassifies deaths that originally have a cause of death that is either a garbage code, a non-specific code (e.g. unspecified stroke), that is assigned to a code that cannot be an underlying cause of death (e.g. senility) or an intermediate but not underlying cause of death, using regression models, redistribution based on fixed proportions and proportion or fractional assignment of a death assigned to multiple causes.

In order to make the information comparable to the causes of death that can be estimated with the PHMRC VA instrument, causes of death were grouped in 34 causes for adults, 21 for children and 6 for newborns. The causes of death were mapped from ICD10 codes using the methodology described in the PHMRC project.

### Analysis

Mortality fractions were compared by cause according to VA and vital statistics for each age group in general, with the stratification between deaths occurring in communities and in medical units. Information was analyzed separately for each age group including neonatal deaths (death in the first 27 days of birth), children (deaths after 28 days and younger than 12 years) and adults (deaths older than 12 years), according to the design proposed by PHMRC. Analyses were performed using the statistical package Stata version 11. [27]

## Results

(Fig 1) shows the process of collecting the VAs. The final sample included 1,201 complete VAs. It is important to highlight the difficulties in locating the homes of informants. In more than 25% of the sample selected, the addresses were incorrect or lacked sufficient information to identify the households for the interview. Due to geographical accessibility problems, 144 (4.6%) cases were not visited. On the other hand, in the case of visited homes where it was certain that the patient lived, the interview was not conducted in 496 cases (19.3%) because the relative who cared for the patient was not home after three visits.

There were 108 refusals to conduct the interview (4.2%). The main reason was not wishing to revisit painful moments. The interview was not conducted in five cases on ethical grounds due to recent mourning. None of the informants requested emotional support and no one required a simultaneous interpreter. Among the 1,201 interviews conducted, supervisory re-interviews were conducted in 291 (24.2%) randomly selected cases.

No statistically significant differences were found when analyzing the differences between the percentage of complete and incomplete interviews by age group and sex. The analysis included a total of 1,198 deaths, and three adult cases were eliminated due to incomplete information in violent deaths. The analysis of certain variables in death certificates (DCs) revealed that 1,011 cases (85.39%) received medical attention before death. Diagnostic autopsy was performed in 114 cases (9.52%). In regard to the place of death, 503 cases (41.99%) died in a health institution (Table 1). In this study, 32.9% of deaths were certified by the physician who was providing care to the deceased person, 17.3% by legal issues physician, 47.6% by other personnel authorized by the Secretary of Health, 1.9% by civil authorities and 0.3% by others. The share of cases in which the basic cause of death in vital statistics corresponded to ill-defined causes was 11.9% in adults, 1.9% in children and 6.9% in neonates.

**Table 1:** General characteristics of deaths in the sample, including information from death certificates Hidalgo, Mexico, 2009.

	Sex		Received medical care before death	Without social security	With necropsy	Place of death			
Age group	Male	Female				Hospital	Public space	Home	Unknown
	644 (53.76%)	554 (46.24%)	1011 (85.39%)	447 (37.31%)	114 (9.52)	503 (41.99%)	64 (5.34%)	589 (49.17)	42 (3.51)
0–28 days	3.11	4.15	4.15	2.24	0.88	7.75	3.13	0.34	0
29 days- 1 year	1.86	0.54	1.29	0.67	0.88	1.99	0	0.85	0
1–4 years	1.55	1.44	1.29	1.57	2.63	1.99	4.68	0.68	2.38
5–11 years	1.55	1.44	1.48	1.79	3.51	2.39	3.13	0.68	0
12–19 years	2.48	0.91	1.29	2.24	10.53	1.79	4.68	0.68	11.9
20–44 years	14.6	10.65	10.09	15.88	42.11	14.51	48.44	7.13	16.67
45–59 years	17.55	14.44	15.92	18.79	18.42	17.69	15.63	13.41	35.72
60+ years	57.3	66.43	64.49	56.82	21.04	51.89	20.31	76.23	33.33

(Fig 2) shows the distribution of deaths examined according to age group and sex, including a bar with the 95% uncertainty interval around the estimate for each CSMF A total of 1,104 deaths were included in the adult group, 51 VA in children and 43 neonatal cases. When analyzing the VAs, the Tariff method for adults managed to determine the cause of death of 972 cases in adults, with only 9% of cases classified as undetermined cause. The highest CSMFs were observed for other non-communicable diseases, diabetes, acute myocardial infarction and cirrhosis. These were also the four main causes of death according to information from death certificates. In general, the CSMFs calculated from VAs were similar to those obtained from DCs after redistribution of ill-defined causes. The causes with the greatest discrepancies were chronic obstructive pulmonary disease, other infectious diseases and stroke.

**Fig 2:**
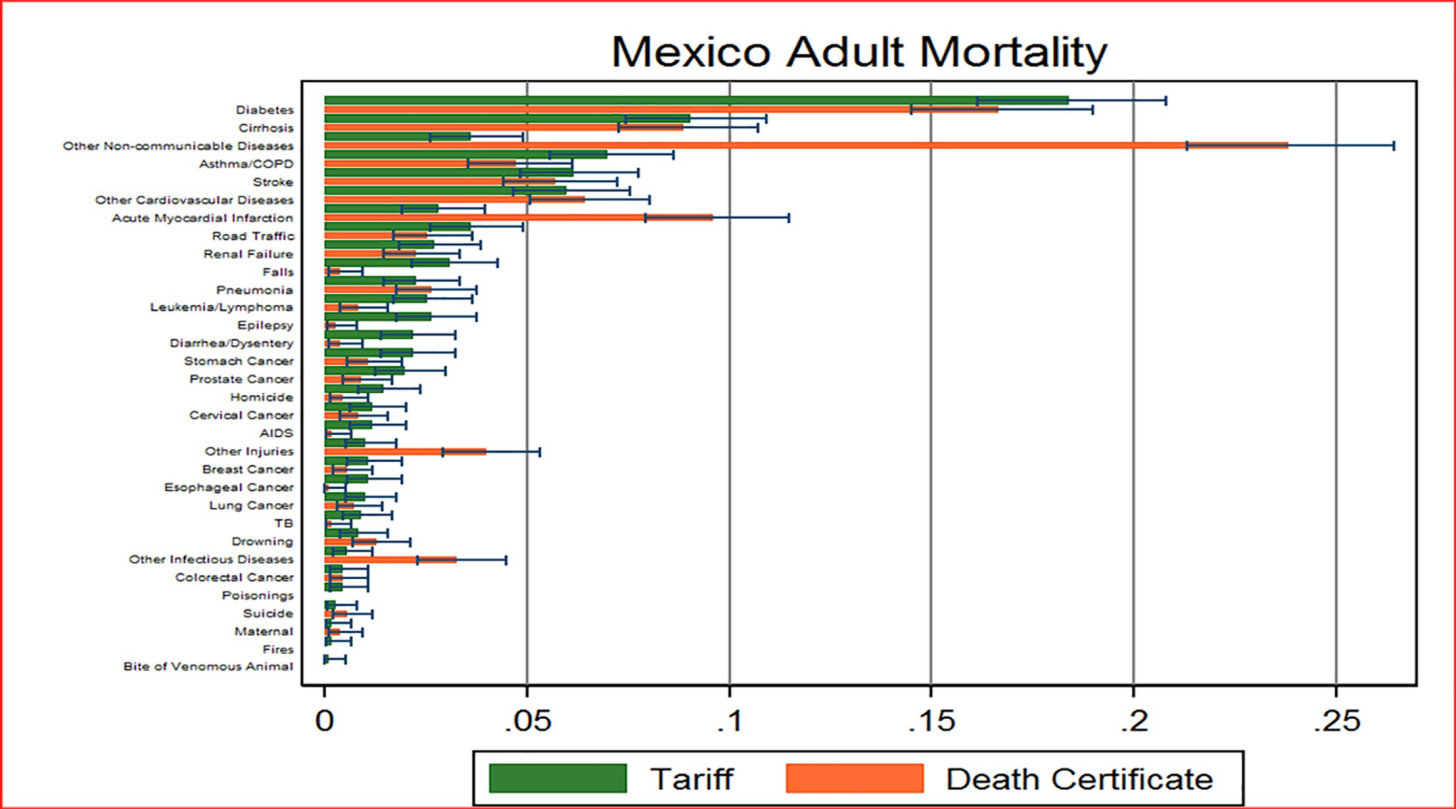
Comparison of adult mortality fraction calculated by VAs vs. vital statistics, Hidalgo, Mexico 2009 (n = 1,104).

(Fig 3) shows the specific fractions of mortality by cause in children. The Tariff method in this age group determined the cause of death in 51 cases, with only 2% of cases classified as undetermined cause. The highest CSMFs were observed for other causes of death and sepsis with local bacterial infection. Pneumonia, other cancers, infections diseases, sepsis and other cardiovascular diseases had discrepancies between the causes of death determined by VA and DCs.

**Fig 3:**
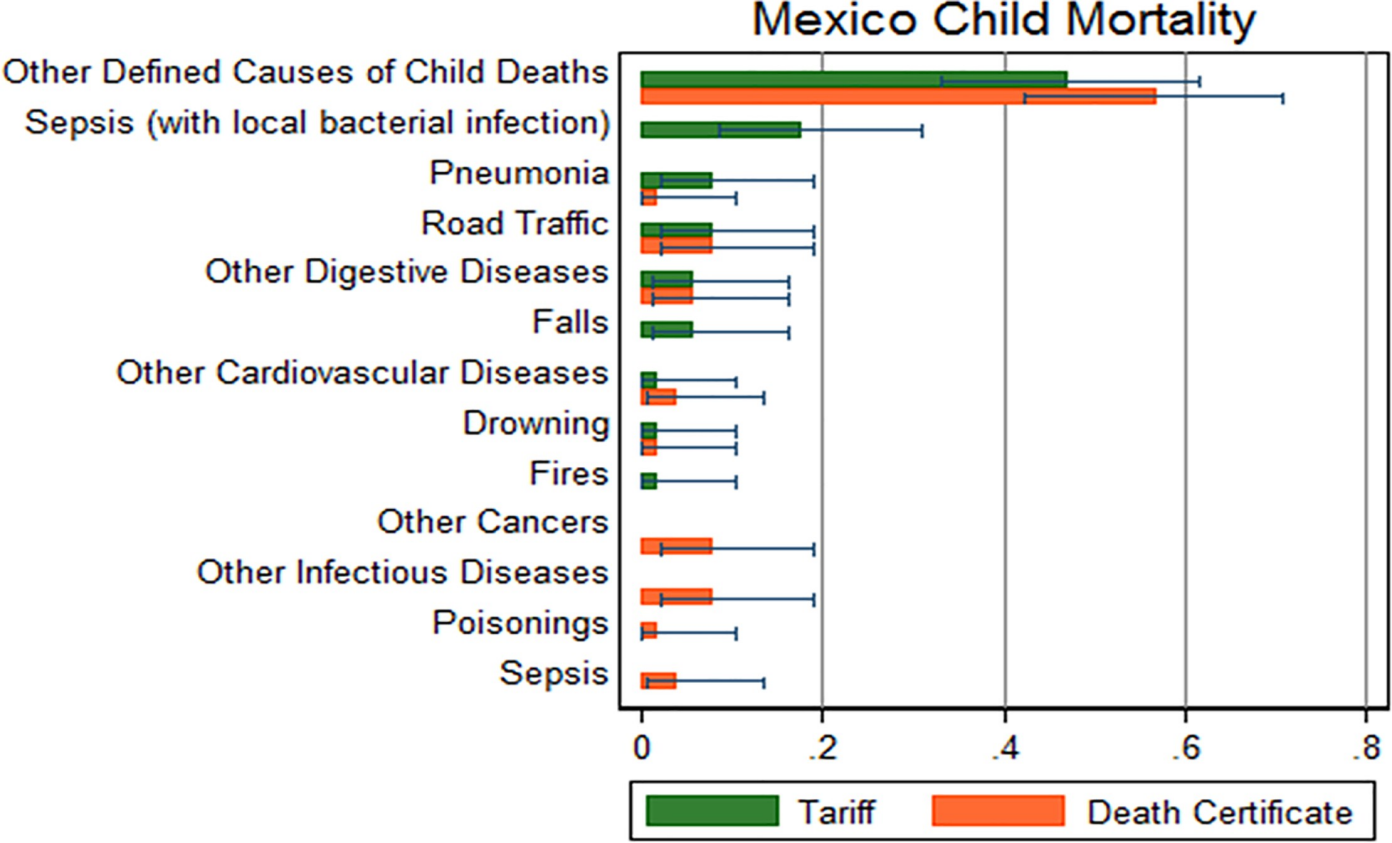
Comparison of child mortality fraction calculated by VAs vs. vital statistics, Hidalgo, Mexico 2009 (n = 1,104).

**Fig 4:**
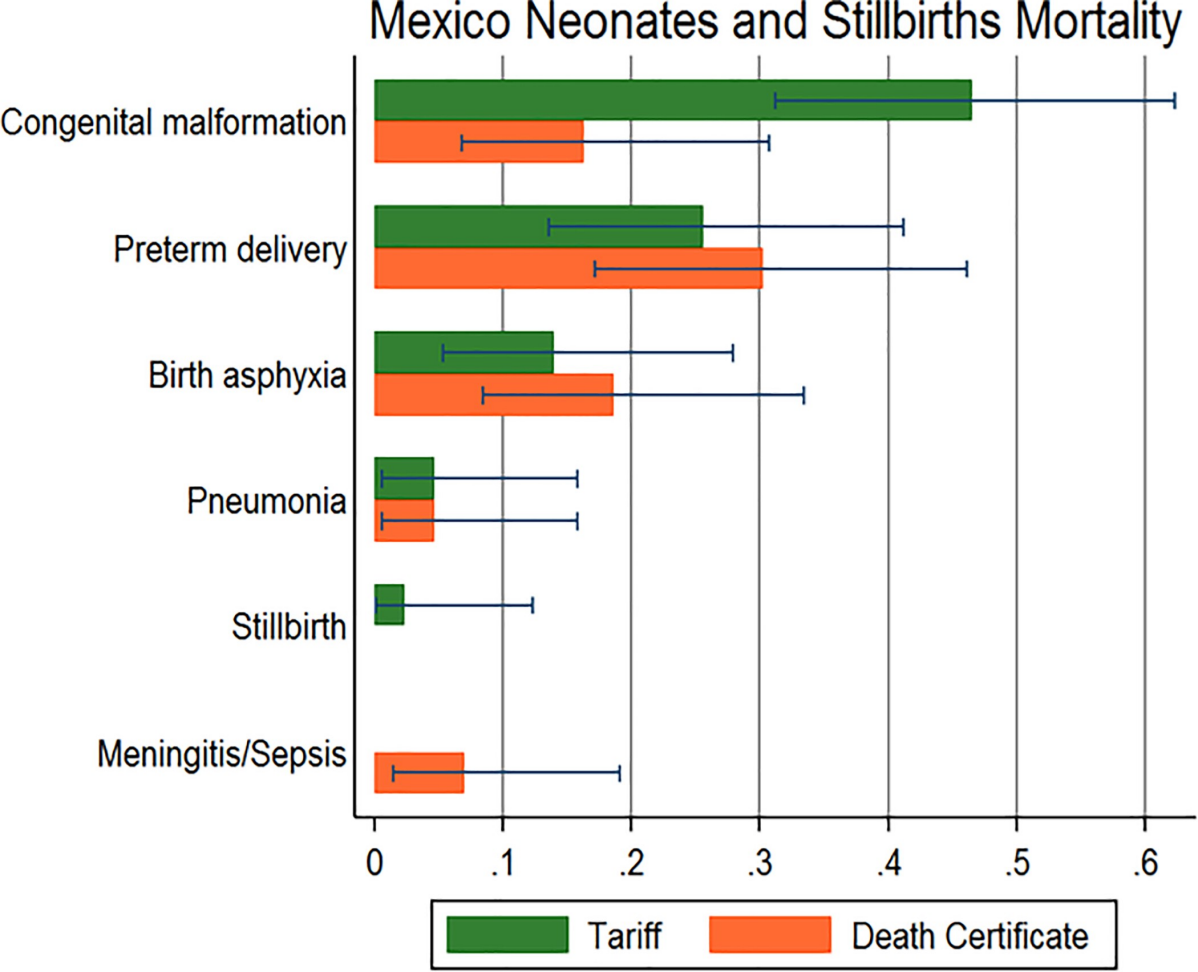
Comparison of neonate mortality fraction calculated by VAs vs. vital statistics, Hidalgo, Mexico 2009 (n = 1,104).

In the neonates group, the Tariff method determined the cause of death in 43 cases, with only 7% of those classified as undetermined cause. The highest CSMFs were observed in congenital malformations, preterm delivery and birth asphyxia. These were also the leading causes of death according to death certificate information (Fig 4).

## Discussion

This study allowed the calculation of CSMFs from VAs analyzed with the Tariff method in deaths occurred in the state of Hidalgo, Mexico. This method made it possible to estimate CSMFs that were similar in many cases to those found in vital statistics.

This study is important for two reasons. First, although several studies have calculated CSMFs from verbal autopsies, [28–30] this is one of the first to make this assessment using the Tariff method on a sample of community deaths. Secondly, it is the first study in Mexico to measure CSMFs at a population level using this methodology.

The PHMRC study developed a VA questionnaire and automated methods for analysis, including the Tariff method. While this study validated the questionnaire and analytical methods with a sample of more than 12,000 hospital deaths with a confirmed cause of death, [29] it had to be tested on community deaths. These deaths may not be as clearly defined as those in hospitals, and informants may have less information about the event of death. However, the Tariff method succeeded in assigning a cause of death to most cases, with low proportions of undetermined cause of death. The use of VAs validated and analyzed with the Tariff method managed to determine mortality fractions by cause similar to those obtained in vital statistics. In the case of adults, the first four causes of death in the list considered in this study are the same from verbal autopsies as in vital statistics (other non-communicable diseases, diabetes, acute myocardial infarction and cirrhosis). On the other hand, there is lower agreement in the CSMFs for causes like stroke, COPD or other infectious diseases. Early analysis of the PHMRC VA questionnaire documented a limited capacity of the questionnaire to identify in an accurate way these causes, with chance corrected concordances between VA and medical records ranging from 10 to 50% in hospital deaths.[21] The results of this study may reflect limitations of the VA to identify these causes of death, but may also reflect problems in the vital registration related to these conditions.

The agreement between the mortality fractions by cause according to these two sources shows some variations for children and neonates, with an overall lower performance as compared to the one we found for adults. On one hand, the small sample size in this age groups may affect our capacity to detect associations between the CSMFs estimated from the VA and vital registration. However, it is important to recognize the limitations in the performance of this VA questionnaire to assess causes of death in children and neonates, which has been documented before. From the early developments of the PHMRC VA instrument, it shown a lower performance at the individual and population level, as documented in the first validation paper using the Tariff method.[21] In fact, this limitation led to a review of the causes of death the questionnaire was able to identify with a minimum level of confidence, reducing the list from 11 to 6 causes of death for neonates. Although the performance of the PHMRC instrument with children and neonates improved with the development of the new Tariff method (which is used in this analysis), it was still lower as compared to the one we saw for adults in an analysis of hospital deaths. [22] The findings of this study, now analyzing data from a community sample, confirm the previous findings with hospital deaths. Possible reasons for this may be the capacity of informants to provide information on symptoms leading to a cause of death in children or neonates, or to the nature of causes in these age groups. It is also possible to have classification errors in vital statistics for children deaths, which limitations have been also documented in Mexico. [17] [31]—More research is required in this area to improve the performance of this method with neonatal and child deaths.

### Limitations

The study has some limitations that should be considered in the interpretation of results. First, the VA questionnaire developed by PHMRC has limited potential to identify causes of death. Therefore, it is only possible to have details for up to 34 causes of death for adults, 21 for children and 10 for newborns. [16]

A significant percentage of cases classified as having poorly defined causes were found when analyzing the classification of causes of death in vital statistics to the deaths included in this study. Provided that the reassignment of ill-defined causes of death in vital statistics is performed at the population level, rather than at the individual level, it was not possible to assess coincidence measures at the individual level between the distribution of causes of death according to VA and vital statistics in this study, but only to compare the CSMFs by both methods.

Also, this study does not have a gold standard to evaluate the performance of the VA. Due to this limitation, mortality fractions obtained from VAs were compared against vital statistics. However, death certificates are not a golden standard for comparison. Previous studies have documented that the correlation between causes of death in death certificates versus hospital records (considered a golden standard) varies from 65% for adults, to 38.5% for children and 54.3% for newborns. [17] For this reason, the comparison between the causes of death of these sources should be treated with caution, since both sources have a margin of error. However, it is important to highlight the concordance between the mortality fractions calculated with both sources despite this limitation.

Finally, the small sample size for child and newborn deaths in this study limits our ability to draw conclusions about these groups. Although Mexico has a high-quality statistical system, specific studies have detected problems in assigning causes of death in vital statistics in Mexico, [17] and a significant portion of the cases included in this study were recorded with ill-defined causes of death. This suggests the possibility of improvement in vital statistics.

VAs have been recognized as a useful tool for calculating CSMFs in places with vital or unreliable statistics. While their use is not intended as a substitute for vital statistics systems, VAs can considerably reinforce these systems by providing information on specific locations or causes.

## Conclusion

Although Mexico has a high-quality statistical system, a significant number of the cases included in this study were recorded with ill-defined causes in vital statistics. Additionally, this led to a redistribution of cases in the study to make a correct comparison, suggesting the possibility of improvement in vital statistics. Verbal autopsies have been recognized as a useful tool for calculating mortality fractions by cause in places with vital or unreliable statistics. While their use is not intended as a substitute for vital statistics systems, VAs can considerably strengthen these systems by providing information on specific locations or causes.

In conclusion, this study shows that the application of VAs and their analysis with the Tariff method is an economic option and provides good performance for calculating mortality fractions by cause. Both the questionnaire and the computer program for analysis with the Tariff method (SmartVA) are available. [24] This technique can be used in Mexico or countries with similar conditions for the analysis of deaths by specific causes, or in specific regions, to gather information on causes of mortality that contribute to decision-making in the health sector.
